# Design and Baseline Evaluation of Social Media Vaping Prevention Trial: Randomized Controlled Trial Study

**DOI:** 10.2196/72002

**Published:** 2025-03-31

**Authors:** William Douglas Evans, Megumi Ichimiya, Jeffrey B Bingenheimer, Jennifer Cantrell, Alexander P D'Esterre, Olivia Pincus, Linda Q Yu, Elizabeth C Hair

**Affiliations:** 1 Prevention and Community Health Milken Institute School of Public Health George Washington University Washington, DC United States; 2 Department of Social and Behavioral Sciences School of Public Health New York University New York, NY United States; 3 Schroeder Institute Truth Initiative Washington, DC United States

**Keywords:** social media, e-cigarettes, randomized controlled trial, nicotine, oral nicotine products, nicotine poly-use

## Abstract

**Background:**

Electronic cigarette (e-cigarette) use is a major public health problem and young adults aged 18-24 years are at high risk. Furthermore, oral nicotine products (ONPs) are growing in popularity in this population. Poly-use is widespread. New methodologies for rigorous online studies using social media have been conducted and shown to reduce nicotine use.

**Objective:**

We report on the design and baseline evaluation of a large-scale social media–based randomized controlled trial to evaluate the effects of antivaping social media on young adult vaping and determinants of use.

**Methods:**

Using the Virtual Lab social media platform, participants were recruited using an artificial intelligence chatbot and social media advertising, completed a baseline survey, and were randomized to 1 of 4 study arms. The design was to achieve specific numbers of impressions per arm over 3 survey time points. We recruited 8437 participants, stratified by vaper (n=5026) and nonvaper (n=3321) status. Questionnaire data were collected using the Qualtrics survey platform. Future analyses will examine the effects of social media content on vaping at the endline. Our data analysis describes the 2 cohort samples, examines balance across the 4 study arms on baseline variables in each of the cohorts, and evaluates the internal consistency of several multi-indicator measures of psychosocial constructs.

**Results:**

Among vapers, almost three-fourths were current vapers, >40% were current smokers (using in the past 30 days), and >48% were current poly-users (using e-cigarettes and ≥1 other tobacco products). Substantial numbers of current vapers also currently use some other product, including cigars (n=1520, 30.2%), hookah (n=794, 15.8%), smokeless devices (n=462, 9.2%), and ONPs (n=578, 11.5%). The average age of participants was 21.2 (SD 2) years. Just less than 45% of participants were non-Hispanic White (n=3728, 44.7%), just less than 47% (n=3913, 46.9%) of the sample was male, more than 44% (n=3704, 44.4%) reported completing high school, and 79.3% reported meeting basic needs or better. There were no significant differences between arms and strata by any of these demographics. We calculated scale scores for depression and covariates related to nicotine use and found high alphas. Finally, participants who reported having seen antitobacco brand advertising were more likely to have higher levels of these variables and scales than participants who reported not having seen the advertisements. These results will be examined in future studies.

**Conclusions:**

Social media can be used as a platform at scale for longitudinal randomized controlled trials over extended periods, which extends previous research on short-term trials. Interventions delivered by social media can be used with large samples to evaluate social media health behavior change interventions. Future studies based on this research will evaluate the intervention and dose-response effects of social media exposure on vaping behavior and determinants.

**Trial Registration:**

ClinicalTrials.gov NCT04867668; https://clinicaltrials.gov/study/NCT04867668

## Introduction

### Vaping and Novel Nicotine Products

Electronic cigarettes (e-cigarettes) have been sold in the United States since 2007 and were the most commonly used tobacco product among young adults aged 18-24 years in the United States between 2014 and 2019 [1]. In 2019, the current use of cigarettes and e-cigarettes was 4.5% and 14% [2] among adults, respectively. Although e-cigarette use (vaping) among this population has decreased in recent years, use prevalence still remains at concerning levels, with people aged 18-24 years having the highest levels (11%) in 2021 [3]. Flavored e-cigarettes are widespread in the e-cigarette marketplace, attracting young adults, and there is a wide variety of disposable e-cigarettes, or single-use e-cigarettes, that do not use prefilled pods containing e-liquid. The latest e-cigarettes also contain some of the highest nicotine levels ever seen in tobacco products [4-7]. The use of e-cigarettes has also been associated with worsened lung health and mental well-being [8-12].

In addition to e-cigarettes, other novel nicotine products are growing in popularity. Cornacchione Ross et al [13] found that approximately 17.1% of youth reported using flavored novel oral nicotine products (ONPs) in the past month. Common products included gum, gummies, and lozenges. The use of these novel products is also associated with the use of other nicotine and tobacco products, including e-cigarette use, little filtered cigars or cigarillos, water pipe tobacco, large cigars, smokeless tobacco, and multiproduct (poly) use [13].

e-Cigarettes and other novel nicotine products are marketed widely on social media. Social influencers (individuals who have large followings and have established credibility in a specific area of interest) frequently post about branded nicotine products and promote other online sites and sources related to product use. Given the near-ubiquitous use of social media by individuals aged 18-24 years [14], these trends represent risk factors for young adults to initiate and establish nicotine product use.

### Social Media Interventions

At the same time, social media is a promising intervention strategy to prevent and control nicotine product use. Digital media, including social media platforms, have become a part of our daily lives, particularly among young adults [14]. Because of its ubiquity and potential for influence, digital media can be a valuable or harmful tool for population-level behavior change. Recent studies have demonstrated the potential for targeted social media campaigns to reduce risk factors for e-cigarette use [15]. Other research has shown that social media using influencer strategies can promote health behaviors and reduce risk behaviors in other domains of public health, such as vaccination [16]. However, there are relatively few large, well-controlled studies on social media as an intervention channel in nicotine and tobacco control, which highlights the need for more research on the relationship between digital media and behavior change, social norms, and social networks [17]. Although research is being conducted to determine what digital media as an intervention tool would look like, how it works, and how effective it is [17], these studies have only scratched the surface [18].

Methods for digital media research for behavior change have advanced substantially in recent years, and digital health interventions have demonstrated effective methods for health promotion [19,20]. In recent years, new methodologies and technologies for delivering interventions and conducting rigorous online studies using social media have been conducted and shown to reduce nicotine use and predictors such as beliefs about product use, social norms, and behavioral intentions [15,21].

These studies, and the emerging evidence, are based on Social Cognitive Theory (eg, modeling healthy behavioral choices to avoid nicotine use [22]) and Social Norms Theory (eg, demonstrating avoidance of nicotine as a norm among adolescents and young adults (AYA) [23,24]). New experiments can demonstrate how to optimize these methods and compete directly with mis- and disinformation promoted by the vaping and tobacco industries and in the future can be applied to new and emerging nicotine products such as Zyn [25].

One area of emerging interest is the science of creating engaging, platform- and population-appropriate content delivered in a realistic manner, and kept fresh and interesting through multiple campaigns on social media (treatment [26]). Public health interventions can learn from corporate social media campaigns by aiming to create engaging content through multiple strategies, including social influencers [27]. This study applies these techniques in the context of a naturalistic social media intervention with young adults to prevent e-cigarette use.

### Current Research

This study builds on previous research by this investigative team that successfully used social media recruitment and intervention delivery. Here we report on a large-scale randomized controlled trial (RCT) on social media intervention to prevent vaping among AYA over an extended time frame and evaluate the effectiveness of social media– based on the content delivered through multiple social media campaigns. We tested three main hypotheses:

1. Hypothesis 1: We can effectively establish a bespoke, large-scale, social media–based longitudinal panel to evaluate antivaping interventions over time (primary hypothesis for this study).
2. Hypothesis 2: Exposure to antivaping social media content will reduce AYA vaping over time.
3. Hypothesis 3: Higher dosage of antivaping social media content will reduce AYA vaping to a greater extent over time (dose-response effect).

Here we report on the design and baseline evaluation of this large-scale, social media–based RCT. Future studies will report on results for hypotheses 2 and 3.

## Methods

### Study Design

This study reports solely on the baseline evaluation of a longitudinal study of vaping among AYA in the United States. The overall study is an RCT (registered at clinicaltrials.gov under NCT04867668) with 3 treatment arms plus a no-exposure control arm. Using the Virtual Lab platform, participants were recruited into the study (details below), completed a baseline survey, and were then randomized to 1 of the 4 study arms. The design was to achieve a specific number of impressions per arm as follows: 0 (control), 8 (low), 16 (medium), and 32 (high) over 3 time points over a planned 9-month period (baseline to endline).

The first treatment period was between baseline data collection and a planned 3-month follow-up (FU1). The second treatment period was between FU1 and a 9-month follow-up (FU2). Impressions are defined as the number of viewings of a social media post by a study participant [28]. The actual duration of treatment varied by participant due to the timing of survey completion, and this variation will be incorporated into future panel analyses.

The overall RCT aim was to collect sufficient participants within each wave to have sufficient power to detect a treatment effect of intervention content exposure on current vaping at FU2. The final baseline sample consisted of 8347 divided into the 4 study conditions. Follow-up samples will be reported in future studies (Multimedia Appendix 1).

### Statistical Power

To determine the necessary sample sizes for the study, we made a series of assumptions. Table 1 represents the expected prevalence of current vaping at endline (FU2) among the two cohorts: (1) current vapers at enrollment and (2) current nonvapers at enrollment. The assumed vaping prevalence of 90% among control group members of the vapers’ cohort reflects the reality that, even in the absence of intervention, a small fraction of vapers will quit on their own; likewise, the 10% vaping prevalence among the nonvaper cohort reflects the assumption that some nonvapers will begin vaping. The endline prevalence assumptions in the low, medium, and high dosage arms represent our assumptions about the extent to which the social media intervention will increase cessation among vapers and decrease initiation among nonvapers and are consistent with findings of other recent studies examining the impacts of social media–based interventions on tobacco use [29].

Using these numbers, we estimated the sample size that would be required to provide 80% power to reject the null hypothesis of no intervention effect using a dose-response type analysis (ie, a monotone trend across the arms rather than a set of group comparisons). Under these assumptions, the required endline sample size was 2474 for the vaping cohort and 1569 for the nonvaping cohort. Finally, we assumed that attrition between enrollment and endline would be 50% for baseline vapers and nonvapers alike, leading to a required sample of 4949 vapers and 3138 nonvapers at enrollment.

**Table 1:** Vaping prevalence by study arm and vaping status.

Study arm (dosage)	Vapers (%)	Nonvapers (%)
None	90	10
Low	88.33	8.33
Medium	86.67	6.67
High	85	5

### Intervention Content and Delivery

One approach to delivering social media–based online interventions and research studies is the Virtual Lab Platform [30]. Virtual Lab is an open-source platform that enables users to use digital marketing, or “retargeting,” to recruit custom audiences of participants based on specific characteristics and online behavior. This enables the creation of bespoke panels of specific audiences, such as young adults within specific age ranges, living in the United States, and engaging in behaviors such as vaping. This technique can be used to deliver intervention content, such as social media posts, including video and other formats, in specific quantities to panel participants in specified dosages over time.

The intervention content consisted of 2 distinct “campaigns” on Facebook (Meta) and Instagram (Meta). The first campaign ran during the period from baseline to FU1 and consisted of two 15-second videos drawn from a previous online Truth Initiative campaign called “Telenovela” and “Deflated” and two 20-second videos created by the investigators. The rationale for creating our own content was to have both branded Truth videos that potentially might be recognizable to the participants, given that they are the priority audience of young adults, and unbranded videos never previously aired. The second campaign ran from FU1 to FU2, and was structured similarly to the first, with participants receiving 3 newly created videos, all approximately 15 seconds in length.

The main themes of the videos were that vaping can be harmful to one’s mental health, raising anxiety and depression, and that avoiding vaping can alleviate these negative feelings. This is consistent with an “anti-industry” countermarking approach to nicotine and tobacco campaigns, which has been used successfully in the past. The campaign was not publicly active during this study [31,32]. We chose this content because it was designed for social media distribution, focused on preventing vaping, and was not in current public distribution.

Following the baseline, videos were promoted in the live Facebook and Instagram feeds of treatment arm participants in a randomized order and combinations in order to achieve the targeted impressions for each arm (ie, an average number of impressions per arm). For example, the “low” exposure arm was designed to get 8 impressions and would receive a randomly ordered assignment of each video one time, the next highest exposure arm (16 impressions) was designed to get the videos in random order 2 times, and so on. The actual number of impressions per group varied due to the time required by participants to complete surveys and the time of the intervention delivery and was measured at the group level due to confidentiality restrictions Facebook and Instagram place on publicly available user data (ie, the exact number of impressions by the individual user is not available, only by study condition). This resulted in the use of a 5-level variable corresponding to the 4 treatment arms of increasing intended impressions (Arm 1=0 impressions, Arm 2=8 impressions, Arm 3=16 impressions, and Arm 4=32 impressions).

The study was implemented by the Virtual Lab, a social media–based data collection and intervention content delivery platform [28]. Virtual Lab is designed to interface with and recruit participants from the Facebook and Instagram platforms, which are very widely used (especially Instagram) among US young adults [14]. Participants were recruited via paid advertising posts on Facebook and Instagram. When a potential participant clicked on a study advertisement post, they were asked a series of screening questions using a Facebook Messenger (FM) chatbot. Eligible participants were 18- to 24-year-old US residents within the stratified subgroups, stratified by ever-vaping status. Participants were asked to provide informed consent and participate in the study through an FM survey delivered by the chatbot. After completing the baseline questionnaire, participants were randomized to the study condition, received any relevant content over time, and invited to complete the follow-up questionnaires.

### Data Collection and Measures

Similar to a previous RCT study reported by Evans et al [15], we worked with Virtual Lab to implement the study and collect data The study team created a Facebook business account called “Digital Health Research” to recruit participants and manage data collection, and a second account, “Consumer Consciousness,” to run the target advertisements on the enrolled participants’ Facebook and Instagram newsfeeds. The recruitment ads were served to people aged 18-24 years and located in the United States. The advertisements used the text, “Take a 15-minute survey, get paid $30” (to reflect the total compensation for completing all 3 surveys). Multiple graphics were used in the advertising posts to attract interest in the study. After participants clicked on the study’s advertisement, they were sent a message via FM inviting them to participate in the study.

Once participants clicked on the recruitment advertisement, they completed a screening survey delivered as a series of individual chats through FM using a Chatbot. The screening survey determined eligibility based on inclusion criteria, including vaping status to achieve our stratification and power requirement for total vapers in the sample. Once participants were deemed eligible, they read an institutional review board (IRB)–approved informed consent statement. After consent, they clicked on a link to begin the study questionnaire.

Future studies based on this research will report on participant engagement and retention methods, which include Chatbot messages inviting participants to take follow-up surveys, and text messages as additional follow-up where needed. Retention will be a major focus of future studies, and an important question to be examined is how attrition in social media studies compares with other data collection modalities.

The questionnaire consisted of 57 items drawn from the tobacco control and campaign evaluation literature. All items were measured on a 5-point agreement scale except where noted (strongly agree to strongly disagree) [33].

Our primary end point was current e-cigarette use in the past 30 days at endline. In addition, we measured secondary outcomes to include future vape intentions, operationalized as the average of responses to 2 items, which were each answered on a 5-point agreement scale: “Thinking about the future, if one of your best friends offered you an e-cigarette/vape (even one or two puffs) in the coming year, would you smoke it?” and “Do you think you will use an e-cigarette/vape (even one or two puffs) in the next year?” An additional secondary end point was anti-industry sentiment, measured as the average of responses to two items, also on a 5-point agreement scale: “Vape companies make me angry” and “I am willing to stand up with others against vape companies.” Our measures of vaping intentions and anti-industry sentiment are both taken from the second follow-up survey. Finally, we examined self-reported advertisement exposure. For each of the 4 advertisements, participants were asked, “Overall, about how many times to do you think you’ve seen this ad? 1-2 times; 3-5 times; more than 5 times.” Responses were recoded to approximate the average value for each category (“Never”= 0, “1-2 times”=1.5, “3-5 times”= 4, “more than 5 times”=6) and an average value across each of these 4 advertisements was calculated in order to generate an average value of reported advertisement exposure. Because we were interested in cumulative exposure, the value for both campaign periods was averaged.

### Data Analysis

Once follow-up data have been collected, our main analysis will examine the dose-response relationship between the study arm (which represents the intensity of exposure to the social media intervention) and the primary end point of current e-cigarette use. The analyses we present here had three primary goals: to describe the two cohort (vaper and nonvaper) samples, to examine balance across the 4 study arms on baseline variables in each of the cohorts, and to evaluate the internal consistency of several multi-indicator measures of psychosocial constructs. We accomplish the first goal simply by computing frequencies and descriptive statistics on the sociodemographic background and baseline behavioral and psychosocial variables in each cohort. For the second goal, within each cohort (vapers and nonvapers) compare the 4 study arms by cross-tabulating the arm with categorical baseline variables and conducting Pearson chi-square tests; and for continuous variables, by obtaining arm-specific means and carrying out one-way ANOVA. For our third analytic objective, for the indicators of each of the 6 multi-indicator measures of psychosocial factors, we obtain item-specific means and SDs, tables of pairwise correlations among the items, and Cronbach alpha. All analyses were carried out using the Stata 18 (StataCorp) software package. Future studies will use imputation techniques to handle missing data for longitudinal analysis as needed.

### Ethical Considerations

This study was reviewed and approved as not greater than minimal risk human subjects research by the George Washington University’s IRB on August 5, 2021 under approval number NCR202837. Through the FM chatbot, participants read an IRB-approved statement informing them about the purposes and nature of the research. By clicking on a button to proceed to the survey, they provided consent to participate. All data used in this study have been deidentified and stored following the IRB-approved procedure to ensure confidentiality. Participants received a US $5 e-gift card via email as compensation for completing the baseline survey (US $10 and US $15 for the FU1 and FU2 surveys, respectively).

## Results

Table 2 provides descriptive statistics for outcome variables collected in the baseline sample. Overall, we successfully met recruitment targets by surveying more than 5000 vapers and more than 3300 nonvapers at baseline. Among vapers, almost three-fourth (n=3750, 74.6%) were current vapers (vaped within the past 30 d), more than 40% were current smokers (n=2055, 40.9% smoked at least one cigarette in the past 30 d), and more than 48% were current poly-users (using e-cigarettes and one or more other tobacco products). In addition, substantial numbers of current vapers also currently use some other product, including cigars (n=1520, 30.2%), hookah (n=794, 15.8%), smokeless devices (n=462, 9.2%), and ONPs (n=578, 11.5%).

There was substantial awareness of antitobacco and nicotine brands (n=1744, 34.7%; aware), and more than 40% agreed with each of the antitobacco messages included in our intervention (n=2365, 47.1% for message 1 and n=2084, 41.5% for message 2). More than 88% of participants mentioned often seeing advertisements or promotions for at least one type of tobacco product (e-cigarettes, cigarettes, little cigars or cigarillos, or smokeless, snus, or chew tobacco) when using the internet (88.6%). Some 39% of participants declared being exposed to advertisements or promotions for all 4 types of tobacco products online.

In addition, we captured scales for multiple hypothesized determinants of tobacco use behavior. These included measures of perceived risk, social acceptability of vaping, anti-industry, and nonvaping identity. In addition, we calculated an antivape scale, which included a summary of perceived risk, social acceptability, anti-industry, independence, and nonvaping identity. Finally, we included a brief form of the Depression Anxiety Stress Scale (DASS). All of these scales had α scores .66 or higher, and all but independence were .77 or higher.

**Table 2:** Outcome descriptive statistics.

Variables and options		Vapers (n=5026)	Nonvapers (n=3321)	Scale Alpha	
Age (years), mean (SD)		21.4 (1.9)	20.8 (2)	—^a^	
**Race and ethnicity, n (%)**					
	Non-Hispanic White	2360 (47)	1368 (41.2)	—	
	Hispanic	1007 (20)	732 (22)	—	
	Non-Hispanic Black	886 (17.6)	458 (13.8)	—	
	Non-Hispanic Asian	378 (7.5)	434 (13.1)	—	
	Other	395 (7.9)	329 (9.9)	—	
**Sex, n (%)**					
	Male	2159 (43)	1754 (52.8)	—	
	Female	2473 (49.2)	1271 (38.3)	—	
	Another identity	36 (0.7)	38 (1.1)	—	
	Nonbinary or transgender	358 (7.1)	258 (7.8)	—	
**Education, n (%)**					
	Completed high school or less	1098 (21.8)	525 (15.8)	—	
	Completed high school diploma or GED^b^	2213 (44)	1491 (44.9)	—	
	Completed associate’s degree or some college	1115 (22.2)	877 (26.4)	—	
	College graduate (eg, BA^c^, BS^d^)	452 (9)	283 (8.5)	—	
	Completed graduate school	138 (2.7)	132 (4)	—	
	Not sure	10 (0.2)	13 (0.4)	—	
**Residence, n (%)**					
	Own	2000 (39.8)	1510 (45.5)	—	
	Rent	3026 (60.2)	1811 (54.5)	—	
**Marital status, n (%)**					
	Single	4246 (84.5)	2728 (82.1)	—	
	Married	687 (13.7)	465 (14)	—	
	Divorced	93 (1.9)	128 (3.9)	—	
**Employment, n (%)**					
	Employed full-time	1307 (26)	672 (20.2)	—	
	Employed part-time	1026 (20.4)	834 (25.1)	—	
	Self-employed	439 (8.7)	451 (13.6)	—	
	Student	993 (19.8)	927 (27.9)	—	
	Unemployed	1261 (25.1)	437 (13.2)	—	
**Income, n (%)**					
	Do not meet basic expenses	1185 (23.6)	507 (15.3)	—	
	Just meet basic expenses	2130 (42.4)	1326 (39.9)	—	
	Meet needs with a little left	1099 (21.9)	930 (28)	—	
	Live comfortably	612 (12.2)	558 (16.8)	—	
**Tobacco use, n (%)**					
	Ever vaped	5026 (100)	0 (0)	—	
	Current vaper	3750 (74.6)	0 (0)	—	
	Ever smoked	3278 (65.2)	179 (5.4)	—	
	Current smoker	2055 (40.9)	90 (2.7)	—	
	Ever tried poly-use (e-cigarette + any other product)	2963 (59)	0 (0)	—	
	Current poly-user (e-cigarette + any other product)	2419 (48.1)	0 (0)	—	
**Tobacco use intentions, n (%)**					
	Vape intention for next year	3125 (62.2)	691 (20.8)	—	
	Smoking intention for next year	2278 (45.3)	576 (17.3)	—	
**Other tobacco use, n (%)**					
	Ever used cigar	2418 (48.1)	166 (5)	—	
	Current use cigar	1520 (30.2)	97 (2.9)	—	
	Ever used hookah	1698 (33.8)	132 (4)	—	
	Current use hookah	794 (15.8)	74 (12.2)	—	
	Ever used smokeless	857 (17.1)	87 (2.6)	—	
	Current use smokeless	462 (9.2)	73 (2.2)	—	
	Ever used oral nicotine	899 (17.9)	78 (2.3)	—	
	Current use of oral nicotine	578 (11.5)	63 (1.9)	—	
**Brand awareness, n (%)**					
	Truth brand awareness	931 (18.5)	666 (20.1)	—	
	Antitobacco brand awareness (Truth, The Real Cost)	1744 (34.7)	1310 (39.4)	—	
**Message agreement, n (%)**					
	Message agreement 1: No one knows the long-term effects of vaping	2365 (47.1)	1253 (37.7)	—	
	Message agreement 2: People who vape are being tested on	2084 (41.5)	1261 (38)	—	
**Exposure to online tobacco advertisements^e^, n (%)**					
	Exposure to online advertisements			—	
	No exposure to online tobacco advertisements	572 (11.4)	694 (20.9)	—	
	Exposed to one type of tobacco product advertisements online	782 (15.6)	486 (14.6)	—	
	Exposed to two types of tobacco product advertisements online	841 (16.7)	477 (14.4)	—	
	Exposed to three types of tobacco product advertisements online	872 (17.3)	374 (11.3)	—	
	Exposed to four types of tobacco product advertisements online	1959 (39)	1290 (38.8)	—	
**Perceived** **risk, mean (SD)**					
	Perceived risk summary score	3.4 (1.1)	3.4 (1.2)	.86	
**Social** **acceptability** **, mean (SD)**					
	Social acceptability summary score	3.1 (1.1)	3.8 (1.1)	.78	
**Anti-industry, n (%)**					
	Strongly disagree	747 (14.9)	418 (12.6)	—	
	Somewhat disagree	675 (13.4)	372 (11.2)	—	
	Neither agree nor disagree	1658 (33)	905 (27.3)	—	
	Somewhat agree	1100 (21.9)	779 (23.5)	—	
	Strongly agree	834 (16.6)	845 (25.4)	—	
	Missing	12 (0.2)	2 (0.1)	—	
**Independence, mean (SD)**					
	Independence summary score	3.3 (1)	3.4 (1.2)	.66	
**Nonvaping identity, n (%)**					
	Strongly disagree	573 (11.4)	434 (13.1)	—	
	Somewhat disagree	666 (13.3)	478 (14.4)	—	
	Neither agree nor disagree	1604 (31.9)	863 (26)	—	
	Somewhat agree	1178 (23.4)	737 (22.2)	—	
	Strongly agree	993 (19.8)	806 (24.3)	—	
	Missing	12 (0.2)	3 (0.1)	—	
**AVS^f^, mean (SD)**					
	AVS summary score	3.2 (0.8)	3.5 (0.9)	—	
**Mental health (DASS^g^), mean (SD)**					
	Depression (summary of DASS 1-3)	3.5 (2.4)	2.8 (2.3)	.82	
	Anxiety (summary of DASS 4-6)	3.5 (2.6)	2.6 (2.3)	.82	
	Stress (summary of DASS 7-8)	2.7 (1.8)	2 (1.7)	.77	
	Mental Health summary score (DASS 1-8)	9.6 (6)	7.5 (5.5)	.90	

^a^Not applicable.

^b^GED: General Educational Development.

^c^BA: bachelor of arts.

^d^BS: bachelor of science.

^e^Types of tobacco products: (1) e-cigarette, (2) cigarettes, (3) little cigars or cigarillos, (4) smokeless, snus, or chew tobacco.

^f^AVS: Antivape Scales; summary of perceived risk, social acceptability, anti-industry, independence, and nonvaping identity.

^g^DASS: Depression Anxiety Stress Scale.

Table 3 provides demographics for the baseline sample, validation of the randomization to study arms, and explains observed differences between arms. The average age of participants was 21.2 (SD 2) years, and there were no significant differences between study arms or between the vaper and nonvaper strata. Just less than 45% of participants were non-Hispanic White (n=3728, 44.7%), 20.1% were Hispanic, 1344 (16.1%) were non-Hispanic Black, and 812 (9.7%) were of Asian descent. There were no significant differences by race and ethnicity. Just less than 47% (n=3913, 46.9%) of the sample was male, 3744 (44.9%) were female, and 8.3% reported another identity or nonbinary and transgender. There were no significant differences by sex.

More than 44% (n=3704, 44.4%) reported completing high school, just less than 58% (n=4837, 57.9%) reported renting their home, 6974 (83.6%) were single, 1979 (23.7%) were employed full-time, and 79.3% reported meeting basic needs or better (with the remainder reporting that they did not meet basic needs). There were no significant differences by any of these demographics.

Multimedia Appendix 2 shows the results of pairwise correlations of the calculated scales with alpha statistics, including subscales for the DASS. All scales achieved acceptable alpha levels [34]. Future studies of longitudinal outcomes in this study will use these scales in multivariate analyses of tobacco use outcomes.

**Table 3:** Demographics and randomization to study arms.

Variables/Options			Total (N=8347)		Vaper cohort (n=5026)													Nonvaper cohort (n=3321)										
					Control (n=1253)		Low dose (n=1306)		Middle dose (n=1250)		High dose (n=1217)		*P* value				Control (n=857)				Low dose (n=794)		Middle dose (n=865)		High dose (n=805)		*P* value	
Age, mean (SD)			21.2 (2)		21.5 (1.9)		21.4 (2)		21.3 (1.9)		21.3 (1.9)		.18				20.9 (2)				20.8 (2.1)		20.8 (2)		20.8 (2)		.91	
**Race/Ethnicity** **, n (%)**															.35													.60
	Non-Hispanic White	3728 (44.7)		603 (48.1)		627 (48)		573 (45.8)		557 (45.8)		—^a^				355 (41.4)				311 (39.2)		363 (42)		339 (42.1)		—		
	Hispanic	1739 (20.8)		259 (20.7)		253 (19.4)		246 (19.7)		249 (20.5)		—				188 (21.9)				188 (23.7)		189 (21.8)		167 (20.7)		—		
	Non-Hispanic Black	1344 (16.1)		209 (16.7)		237 (18.1)		240 (19.2)		200 (16.4)		—				118 (13.8)				113 (14.2)		128 (14.8)		99 (12.3)		—		
	Non-Hispanic Asian	812 (9.7)		85 (6.8)		99 (7.6)		99 (7.9)		95 (7.8)		—				120 (14)				96 (12.1)		108 (12.5)		110 (13.7)		—		
	Other	724 (8.7)		97 (7.7)		90 (6.9)		92 (7.4)		116 (9.5)		—				76 (8.9)				86 (10.8)		77 (8.9)		90 (11.2)		—		
**Gender** **, n (%)**															.07													.83
	Male	3913 (46.9)		542 (43.3)		539 (41.3)		571 (45.7)		507 (41.7)		—				459 (53.6)				417 (52.5)		456 (52.7)		422 (52.4)		—		
	Female	3744 (44.9)		604 (48.2)		653 (50)		582 (46.6)		634 (52.1)		—				329 (38.4)				305 (38.4)		329 (38)		308 (38.3)		—		
	Another identity	74 (0.9)		12 (1)		10 (0.8)		10 (0.8)		4 (0.3)		—				9 (1.1)				11 (1.4)		13 (1.5)		5 (0.6)		—		
	Nonbinary and transgender	616 (7.4)		95 (7.6)		104 (8)		87 (7)		72 (5.9)		—				60 (7)				61 (7.7)		67 (7.7)		70 (8.7)		—		
**Education** **, n (%)**															.07													.75
	Completed high school or less	1623 (19.4)		267 (21.3)		284 (21.7)		264 (21.1)		283 (23.3)		—				147 (17.2)				130 (16.4)		115 (13.3)		133 (16.5)		—		
	Completed high school diploma or GED	3704 (44.4)		534 (42.6)		567 (43.4)		604 (48.3)		508 (41.7)		—				374 (43.6)				348 (43.8)		416 (48.1)		353 (43.9)		—		
	Completed associate's degree or some college	1992 (23.9)		297 (23.7)		298 (22.8)		254 (20.3)		266 (21.9)		—				223 (26)				215 (27.1)		229 (26.5)		210 (26.1)		—		
	College graduate (e.g., BA, BS)	735 (8.8)		117 (9.3)		118 (9)		96 (7.7)		121 (9.9)		—				75 (8.8)				66 (8.3)		72 (8.3)		70 (8.7)		—		
	Completed graduate school	270 (3.2)		34 (2.7)		38 (2.9)		32 (2.6)		34 (2.8)		—				36 (4.2)				32 (4)		28 (3.2)		36 (4.5)		—		
	Not sure	23 (0.3)		4 (0.3)		1 (0.1)		0		5 (0.4)		—				2 (0.2)				3 (0.4)		5 (0.6)		3 (0.4)		—		
**Residence** **, n (%)**															.73													.44
	Own	3510 (42.1)		495 (39.5)		519 (39.7)		513 (41)		473 (38.9)		—				409 (47.7)				357 (45)		380 (43.9)		364 (45.2)		—		
	Rent	4837 (57.9)		758 (60.5)		787 (60.3)		737 (59)		744 (61.1)		—				448 (52.3)				437 (55)		485 (56.1)		441 (54.8)		—		
**Marital status** **, n (%)**															.40													.54
	Single	6974 (83.6)		1061 (84.7)		1095 (83.8)		1053 (84.2)		1037 (85.2)		—				698 (81.4)				656 (82.6)		706 (81.6)		668 (83)		—		
	Married	1152 (13.8)		172 (13.7)		191 (14.6)		165 (13.2)		159 (13.1)		—				132 (15.4)				106 (13.4)		118 (13.6)		109 (13.5)		—		
	Divorced	221 (2.6)		20 (1.6)		20 (1.5)		32 (2.6)		21 (1.7)		—				27 (3.2)				32 (4)		41 (4.7)		28 (3.5)		—		
**Employment** **, n (%)**															.52													.53
	Employed full-time	1979 (23.7)		332 (26.5)		359 (27.5)		330 (26.4)		286 (23.5)		—				161 (18.8)				182 (22.9)		167 (19.3)		162 (20.1)		—		
	Employed part-time	1860 (22.3)		267 (21.3)		259 (19.8)		259 (20.7)		241 (19.8)		—				223 (26)				208 (26.2)		215 (24.9)		188 (23.4)		—		
	Self-employed	890 (10.7)		102 (8.1)		111 (8.5)		112 (9)		114 (9.4)		—				113 (13.2)				96 (12.1)		131 (15.1)		111 (13.8)		—		
	Student	1920 (23)		235 (18.8)		252 (19.3)		237 (19)		269 (22.1)		—				247 (28.8)				204 (25.7)		241 (27.9)		235 (29.2)		—		
	Unemployed	1698 (20.3)		317 (25.3)		325 (24.9)		312 (25)		307 (25.2)		—				113 (13.2)				104 (13.1)		111 (12.8)		109 (13.5)		—		
**Income** **, n (%)**															.80													.18
	Do not meet basic expenses	1692 (20.3)		302 (24.1)		317 (24.3)		279 (22.3)		287 (23.6)		—				127 (14.8)				122 (15.4)		129 (14.9)		129 (16)		—		
	Just meet basic expenses	3456 (41.4)		533 (42.5)		550 (42.1)		538 (43)		509 (41.8)		—				357 (41.7)				308 (38.8)		365 (42.2)		296 (36.8)		—		
	Meet needs with a little left	2029 (24.3)		253 (20.2)		286 (21.9)		282 (22.6)		278 (22.8)		—				219 (25.6)				222 (28)		235 (27.2)		254 (31.6)		—		
	Live comfortably	1170 (14)		165 (13.2)		153 (11.7)		151 (12.1)		143 (11.8)		—				154 (18)				142 (17.9)		136 (15.7)		126 (15.7)		—		

^a^Not applicable.

Table 4 provides a bivariate analysis of differences in outcome variables and scales by previous exposure to branded antitobacco advertising, such as the Truth campaign and the US Food and Drug Administration’s Real Cost. Overall, we observed a pattern of differences within both the vaper and nonvaper strata in tobacco use, intentions, message 2 agreement (not message 1), and each of the calculated scales. Participants who reported having seen antitobacco brand advertising were more likely to have higher levels of these variables and scales than participants who reported not having seen the advertising. These results are consistent with selective attention bias theory [35]. In this instance, we hypothesize that individuals who vape and use other tobacco and nicotine products are more likely to attend to antitobacco advertising, as observed in previous research [36,37]. These differences in selective attention will be addressed and controlled for in future longitudinal analyses in this study.

**Table 4:** Differences by previous exposure.

Variables and options		Vaper cohort (n=5026)					Nonvaper cohort (n=3321)				
		Sample size (n=5026)	Have not seen antitobacco brands (n=3282)	Have seen antitobacco brands (n=1744)	*P* value	Total (n=3321)		Have not seen antitobacco brands (n=2011)		Have seen antitobacco brands (n=1310)	*P* value
**Tobacco use, n (%)**											
	Ever vaped	5026 (100)	3282 (100)	1744 (100)	—^a^	0		0	0		—
	Current vaper	3750 (74.6)	2355 (71.8)	1395 (80)	<.001	0 (0.0)		0	0		—
	Ever smoked	3278 (65.2)	2223 (67.7)	1055 (60.5)	<.001	179 (5.4)		121 (6)	58 (4.4)		.047
	Current smoker	2055 (40.9)	1393 (42.4)	662 (38)	.002	90 (2.7)		61 (3)	29 (2.2)		.16
**Tobacco use intentions, n (%)**											
	Vape intention for next year	3125 (62.2)	2001 (61)	1124 (64.4)	.02	691 (20.8)		311 (15.5)	380 (29)		<.001
	Smoking intention for next year	2278 (45.3)	1498 (45.6)	780 (44.7)	.53	576 (17.3)		271 (13.5)	305 (23.3)		<.001
**Other tobacco use, n (%)**											
	Ever used cigar	2418 (48.1)	1632 (49.7)	786 (45.1)	.002	166 (5)		117 (5.8)	49 (3.7)		.007
	Current use cigar	1520 (62.9)	1041 (63.8)	479 (60.9)	.17	97 (58.4)		65 (55.6)	32 (65.3)		.24
	Ever used hookah	1698 (33.8)	1203 (36.7)	495 (28.4)	<.001	132 (4)		92 (4.6)	40 (3.1)		.028
	Current use hookah	794 (46.8)	533 (44.3)	261 (52.7)	.002	74 (56.1)		46 (50)	28 (70)		.033
	Ever used smokeless	857 (17.1)	554 (16.9)	303 (17.4)	.66	87 (2.6)		43 (2.1)	44 (3.4)		.031
	Current use smokeless	462 (53.9)	288 (52)	174 (57.4)	.13	73 (83.9)		33 (76.7)	40 (90.9)		.072
	Ever used oral nicotine	899 (17.9)	533 (16.2)	366 (21)	<.001	78 (2.3)		36 (1.8)	42 (3.2)		.008
	Current use of oral nicotine	578 (64.3)	326 (61.2)	252 (68.9)	.02	63 (80.8)		24 (66.7)	39 (92.9)		.003
**Message agreement, n (%)**											
	Message agreement 1: No one knows the long-term effects of vaping	2365 (47.1)	1524 (46.4)	841 (48.2)	.23	1253 (37.7)		781 (38.8)	472 (36)		.10
	Message agreement 2: People who vape are being tested on	2084 (41.5)	1293 (39.4)	791 (45.4)	<.001	1261 (38)		735 (36.5)	526 (40.2)		.04
**Perceived risk**											
	Perceived risk summary score	3.4 (1.1)	3.4 (1.1)	3.4 (1)	.005	3.4 (1.2)		3.7 (1.1)	3 (1.3)		<.001
**Social acceptability**											
	Social acceptability summary score	3.1 (1.1)	3.2 (1.1)	3.1 (1.1)	.005	3.8 (1.1)		3.9 (1.1)	3.6 (1.1)		<.001
**Anti-industry, n (%)**											
	Anti-industry	—	—	—	<.001	—		—	—		<.001
	Strongly disagree	747 (14.9)	532 (16.2)	215 (12.3)	—	418 (12.6)		179 (8.9)	239 (18.2)		—
	Somewhat disagree	675 (13.4)	417 (12.7)	258 (14.8)	—	372 (11.2)		194 (9.6)	178 (13.6)		—
	Neither agree nor disagree	1658 (33)	1162 (35.4)	496 (28.4)	—	905 (27.3)		590 (29.3)	315 (24)		—
	Somewhat agree	1100 (21.9)	655 (20)	445 (25.5)	—	779 (23.5)		472 (23.5)	307 (23.4)		—
	Strongly agree	834 (16.6)	506 (15.4)	328 (18.8)	—	845 (25.4)		575 (28.6)	270 (20.6)		—
	Missing	12 (0.2)	10 (0.3)	2 (0.1)	—	2 (0.1)		1 (0)	1 (0.1)		—
**Independence, n (%)**											
	Independence summary score	3.3 (1)	3.3 (1)	3.3 (1)	.046	3.4 (1.2)		3.6 (1.1)	3.1 (1.3)		<.001
**Nonvaping identity, n (%)**											
	Nonvaping identity				.04						<.001
	Strongly disagree	573 (11.4)	397 (12.1)	176 (10.1)	—	434 (13.1)		177 (8.8)	257 (19.6)		—
	Somewhat disagree	666 (13.3)	418 (12.7)	248 (14.2)	—	478 (14.4)		223 (11.1)	255 (19.5)		—
	Neither agree nor disagree	1604 (31.9)	1072 (32.7)	532 (30.5)	—	863 (26)		553 (27.5)	310 (23.7)		—
	Somewhat agree	1178 (23.4)	751 (22.9)	427 (24.5)	—	737 (22.2)		497 (24.7)	240 (18.3)		—
	Strongly agree	993 (19.8)	634 (19.3)	359 (20.6)	—	806 (24.3)		558 (27.7)	248 (18.9)		—
	Missing	12 (0.2)	10 (0.3)	2 (0.1)	—	3 (0.1)		3 (0.1)	0		—
**Antivape scales (AVS)^b^**											
	AVS summary score	3.2 (0.8)	3.2 (0.8)	3.3 (0.8)	.008	3.5 (0.9)		3.6 (0.9)	3.2 (1)		<.001

^a^ Not applicable.

^b^Antivape scales (AVS): summary of perceived risk, social acceptability, anti-industry, independence, and nonvaping identity.

## Discussion

### Main Findings

Building on previous research [15], this large RCT shows that social media can be used as a platform at scale for longitudinal studies over extended periods of time. We successfully recruited a fully-powered sample of US-based AYAs, stratifying by specific health-related inclusion criteria (vaping), and engaged them in a social media–based prevention intervention. This lays the groundwork for the completion of the current longitudinal clinical trial and opens opportunities for similar social media–based trials in nicotine and tobacco control, and other areas of public health [38].

In particular, this study demonstrates that bespoke panels with specific inclusion criteria and priority populations can be created and tracked to evaluate the impact of interventions. By studying dose-response effects, and differential attention to intervention content [35], the ongoing longitudinal follow-up under this research will analyze the effects on vaping behavior change and determinants of behavior in a large sample. In particular, future analyses on longitudinal data from this project will examine whether higher dosages of social media content can reduce e-cigarette use and whether these effects vary by vaper status and other variables, such as sociodemographic subgroups [39].

In addition, interventions based on retargeting technology, which have been successful in smaller pilot studies, can be used with large samples to evaluate social media health behavior change interventions over time. Given the high reach and intensity of pronicotine content on social media, including for novel products such as ONPs, the opportunity to countermarket these negative health influences, especially on vulnerable populations such as AYA, is a public health priority. The effects of nicotine product use on mental health outcomes, such as depression measured in this study, are critical to understand. Mental health can both be a focus of intervention content (eg, harmful mental health effects as counterarguments against product use) as well as an outcome to evaluate (eg, the protective effects of antivaping social media on reducing depression) [40].

We found that some 3-quarters of the vaper stratum were current (past 30 d) vapers, more than 40% were current smokers, and more than 48% were current poly-users (vaping and at least one other tobacco product). Other products included various combustible and smokeless products, as well as ONPs. The high levels of current use and poly-use will be important outcomes in examining follow-up studies.

We found substantial (generally more than 40%) awareness of antitobacco and antivaping online content, as well as high levels of awareness of vaping and tobacco product content. These are important covariates that may moderate the effects of our intervention and will be examined in future analyses.

Further, we measured a number of scales for multiple hypothesized determinants of tobacco use behavior, including risk, acceptability, industry beliefs, nonvaping identity, and a short form of the DASS, and these all showed high scale alpha scores, making them suitable for future outcome analyses at follow-up.

We also found differences between vaper and nonvaper strata in tobacco use, intentions, some agreement with antivaping messages, and each of the calculated scales. There was an observed selective attention effect for participants who reported having seen antitobacco brand advertising. In other words, these participants were more likely to report higher levels of these variables and scales than participants who reported not having seen the advertising [35]. We will control for potential selective attention bias in future analyses.

Future studies based on this research will evaluate the intervention effects of social media exposure on vaping behavior and determinants such as intentions, social norms, and e-cigarette industry attitudes. IIn addition, future research will include study designs, measurement, and analysis of poly-use behaviors (eg, vaping, smoking, and novel products such as oral nicotine pouches), as well as evaluation of social media content designed. Qualitative research and mixed methods approaches could explore motivations behind product use, and poly-use, to enhance rigorous quantitative studies. Finally, pronicotine use content on social media is a potentially powerful influence on use, and future studies will seek to document exposure to this content as well as evaluate social media and other strategies to countermarket its effects [41].

This study has some limitations. First, the data were collected by convenience through social media. While there is no evidence that this methodology introduces bias into the sample, it should be noted that the data are not nationally representative of AYA. Future studies should examine methods to improve and analyze the representativeness and potential bias of social media–based samples. In addition, the effectiveness of the intervention depends on the algorithms of Facebook and Instagram, which may vary in how they deliver content to different users given their online behavior. This could lead to unintended variations in exposure among participants. We will have data on exposure at the study arm level and will examine this in future studies. Since we do not as yet have full longitudinal follow-up data to report, the full implications and impact of the intervention are not yet known. Also, there are potential confounding effects of antivaping interventions, such as pronicotine peer influences and social media content [42]. Finally, we observed evidence of selective attention among vapers to intervention content, consistent with previous research. This is an important potential source of potential bias to be explored and controlled for in future analysis of the current study’s longitudinal data.

### Conclusions

Social media–based intervention research at scale is a promising approach to nicotine and tobacco programs. The ability to create bespoke social media panel studies using RCT designs creates opportunities to study a wide variety of public health messaging interventions, and health risks, among specific populations. The potential to follow populations, such as young adults, who are high users of social media creates opportunities to study the long-term effects of exposure to online content on health outcomes [43]. Future applications in other areas of health behavior change should be explored.

## Appendix

Multimedia Appendix 1 CONSORT-eHealth checklist (V 1.6.1).

Multimedia Appendix 2 Scales.

## Abbreviations

AYA: adolescents and young adults
DASS: Depression Anxiety Stress Scale
e-Cigarette: electronic cigarette
FM: Facebook Messenger
IRB: institutional review board
ONP: oral nicotine product
RCT: randomized controlled trial
