# Supplementary material for: Design and Baseline Evaluation of Social Media Vaping Prevention Trial: Randomized Controlled Trial Study
Source: J Med Internet Res. 2025 Mar 31;27:e72002. doi: 10.2196/72002 (PMC11997523; doi:10.2196/72002)
Supplement: Multimedia Appendix 2 [file jmir_v27i1e72002_app2.docx]

Multimedia Appendix 2.

Scales.

| Scale reliability and validity | | | | |  | |
| --- | --- | --- | --- | --- | --- | --- |
| **Statistics for cognitive scales: Perceived Risk (α=.86)** | | | | | |  |
|  | **Perceived risk** | | 1 | 2 | | 3 |
|  |  | Risk 1 (Feel bad physically) | —^a^ | — | | — |
|  |  | Risk 2 (Feel bad mentally) | 0.7^b^ | — | | — |
|  |  | Risk 3 (Develop brain changes) | 0.61^b^ | 0.69^b^ | | — |
| Observation, n | | | 8347 | 8347 | | 8347 |
| Mean (SD) | | | 3.29 (1.3) | 3.38 (1.29) | | 3.54 (1.3) |
| **Statistics for cognitive scales: Social Acceptability** **(α=.78)** | | | | | | |
|  | **Social acceptability** | | 1 | 2 | | — |
|  |  | Social acceptability 1 (Social) | — | — | | — |
|  |  | Social acceptability 2 (Not addicted) | 0.64^b^ | — | | — |
| Observation, n | | | 8333 | 8332 | | — |
| Mean (SD) | | | 3.38 (1.23) | 3.43 (1.29) | | — |
| **Statistics for Cognitive Scales: Independence (α=.66)** | | | | | |  |
|  | **Independence** | | 1 | 2 | | — |
|  |  | Independence 1 (Show independence) | — | — | | — |
|  |  | Independence 2 (In control) | 0.5^b^ | — | | — |
| Observation, n | | | 8336 | 8336 | | — |
| Mean (SD) | | | 3.13 (1.25) | 3.51 (1.31) | | — |
| **Statistics for Cognitive Scales: Depression** **(DASS^c^ subscale) (α=.82)** | | | | | | |
|  | **Depression** | | 1 | 2 | | 3 |
|  |  | DASS 1 (Nothing to look forward to) | — | — | | — |
|  |  | DASS 2 (Felt down-hearted) | 0.65^b^ | — | | — |
|  |  | DASS 3 (Not enthusiastic) | 0.54^b^ | 0.63^b^ | | — |
| Observation, n | | | 8340 | 8341 | | 8342 |
| Mean (SD) | | | 1.11 (0.94) | 1.15 (0.93) | | 0.95  (0.91) |
| **Statistics for Cognitive Scales: Anxiety** **(DASS subscale) (α=.82)** | | | | | | |
|  | **Anxiety** | | 1 | 2 | | 3 |
|  |  | DASS 4 (Worried) | — | — | | — |
|  |  | DASS 5 (Close to panic) | 0.64^b^ | — | | — |
|  |  | DASS 6 (Felt scared) | 0.54^b^ | 0.64^b^ | | — |
| Observation, n | | | 8334 | 8337 | | 8336 |
| Mean (SD) | | | 1.12 (1) | 1.04 (0.97) | | 0.98  (0.97) |
| **Statistics for Cognitive Scales: Stress** **(DASS subscale) (α=.77)** | | | | | | |
|  | **Stress** | | 1 | 2 | | — |
|  |  | DASS 7 (Used nervous energy) | — | — | | — |
|  |  | DASS 8 (Difficult to relax) | 0.62^b^ | — | | — |
| Observation, n | | | 8337 | 8328 | | — |
| Mean (SD) | | | 1.17 (0.99) | 1.25 (0.1) | | — |

^a^Not applicable.

^b^*P*<.001.

^c^DASS: Depression Anxiety Stress Scale.
